# A Closed-form Expression to Estimate the Uncertainty of THD Starting from the LPIT Accuracy Class

**DOI:** 10.3390/s20061804

**Published:** 2020-03-24

**Authors:** Alessandro Mingotti, Lorenzo Peretto, Roberto Tinarelli

**Affiliations:** Department of Electrical, Electronic and Information Engineering—Guglielmo Marconi Alma Mater Studiorum—University of Bologna, Viale del Risorgimento 2, 40136 Bologna, Italy; lorenzo.peretto@unibo.it (L.P.); roberto.tinarelli3@unibo.it (R.T.)

**Keywords:** THD, accuracy class, instrument transformers, sensors, probability density function, variance, probability distribution

## Abstract

Power quality is a wide-ranging and current topic that involves a huge effort from the scientific community. Power quality issues have to be avoided or solved in order to preserve the integrity of the network and its assets. To this purpose, several power quality indexes and measurement techniques have been developed and used by experts. This paper aims at solving the issue of having an uncertainty associated to the total harmonic distortion (THD) measurements. The idea is to obtain a close-form expression, which only requires the knowledge of the instrument transformer accuracy class, to estimate the mean value and the variance of THD. After the development of such an expression, it has been tested and stressed to confirm its effectiveness and applicability in a variety of conditions, and for harmonics up to 25^th^ (of 50 Hz), defined by the standards.

## 1. Introduction

The classical structure of the power network underwent a huge change after the introduction of several new actors. With particular emphasis on medium and low voltage networks (MV and LV), such new actors include: electronic converters, renewable energy sources, measurement instruments, charging stations for electric vehicles, and storage systems. All have contributed to the improvement of the power network towards the so-called and well-known smart grids.

The integration of these new actors brought benefits to both the costumers and distribution system operators (DSOs), which can manage the network in a smarter way, gathering all the necessary information to control each aspect of the grid. However, the new smart grids are not problem-free. As a matter of fact, two of the main issues affecting the network are (i) the reliability of the electrical assets and (ii) the behavior of the network and of assets in the presence of low power quality (PQ).

The former issue concerns the reliability of the existing or new assets when operating at the new and current conditions of the network. To this purpose, the literature provides a variety of works tackling almost all particular aspects related to the assets. For example, a critical cable accessory like the cable joint is tackled in [1,2,3,4,5], while studies on electrical insulators have been completed in [6,7,8,9]. Two asset management systems have been developed in [10,11] and the integration of electric vehicles and storage systems into smart grids has been detailed in [12] and [13], respectively. Lastly, the performance of energy meters under non-rated conditions, hence affected by PQ issues, are studied in [14,15,16,17,18,19].

Turning to the effects of PQ on the network, it is well documented that a low level of PQ may compromise the operation of the network and of its assets. For example, voltage dips effects have been discussed for wind turbines and storage systems in [20] and [21], respectively. In [22,23,24,25,26], techniques to mitigate the effects of harmonics and to improve PQ for converters/inverters have been presented. Finally, crucial assets that must work in all possible network conditions are the instrument transformers (ITs). Their correct behavior is crucial for different purposes, (i) measurements for billing [27,28,29], (ii) measurements to feed management and control algorithms for DSOs and utilities [30,31,32,33,34,35,36], (iii) knowledge of the network parameters. Consequently, studies on ITs behavior at non-rated conditions, including effects of a low PQ, are always vivid and current. In [37,38,39] effects of the harmonics and mitigation techniques have been developed for current transformers (CTs), while the same issue has been tackled in [40,41] for voltage transformers (VTs). A comparison among MV VTs, for PQ purposes has been completed in [42], while the effects of modulation on CTs has been studied in [43].

In light of the above, this paper aims at providing a closed-form expression to evaluate the uncertainty associated to an important parameter like the total harmonic distortion (THD). In fact, considering the complexity of its expression, it is typically measured without any information regarding the associated uncertainty. This results in inaccurate, or in some cases meaningless, information provided to the final user and to DSOs. The literature related to this particular aspect is not wide; however, in [44] an approach for the THD’s uncertainty evaluation based on the guide for the expression of uncertainty on measurements (GUM) [45] has been presented. The THD uncertainty is studied in [46] starting from a characterization of the instrumentation. Finally, [47] assessed the uncertainty of several PQ parameters from a probabilistic point of view and [48] provides a complete overview of the issues related to the measurements of the PQ indices, respectively.

The novelty of this work, as detailed in the following sections, is to provide a closed-form expression of the uncertainty related to THD, which only requires the accuracy class information of the low-power instrument transformers (LPITs) involved for the measurement of the voltages. Therefore, even non-experts or DSOs operators may evaluate the uncertainty related to their THD measurements.

What follows has been structured as: Section 2 provide a brief overview of the PQ scenario and the related standards. Section 3 details the developed expression and all the mathematical steps involved. In Section 4, the performed tests and the obtained results are discussed to verify the effectiveness and applicability of the THD uncertainty expression. Finally, Section 5 collects the main conclusion and significant comments on the work.

## 2. Power Quality Overview

Even if the original meaning was related to voltage, PQ is defined as “characteristics of the electric current, voltage and frequencies at a given point in an electric power system, evaluated against a set of reference technical parameters”, according to the International Electrotechnical Commission vocabulary [49].

From a DSO perspective, a reference standard is the EN 50160 [50], in which continuous phenomena and voltage events that may affect the network are described for all voltage levels. Furthermore, limits for the voltage, frequency and harmonics during the normal operation of the network are fixed. In particular, for the voltage and the frequency is established that they should not vary more than ±10% and ±1% of their rated values, respectively. As for the harmonics from the 2^nd^ to the 25^th^, aim of the work, the limits defined in [50] are listed in Table 1. Higher harmonic orders are not tackled by the standard due to their unpredictable behavior and low amplitude compared to the 50, 60 Hz component.

In addition to the limit fixed in the table, the standard [50] imposes that the THD of the supply voltage should be less than 8% for whatever combination of harmonics affecting the supply voltage (up to the 40^th^).

Another fundamental standard for DSO and power network owners is the IEC Std 519-2014 [51], which provides recommended practice and requirements for harmonic control in electric power systems. The standard purpose is to define goals for system designers who have to build electrical power systems that include both linear and non-linear loads. In the document, after the relevant definitions and equations, the current and voltage distortions limits at the point of common coupling (PCC)—for different voltage and current levels—are given.

Turning to another perspective, the reference standards for manufacturers and final users are the IEC 61000-4-7 [52], the IEC 61000-4-30 [53], and the IEC 61869 series. In [52], instrument manufacturers are asked to build their devices fulfilling some measurement requirements for the voltage, current, and power. These requirements are fixed in terms of error on the rated values (for voltage, current, and power) and in terms of percentage variation for the harmonic measurements. In [53], instead, is a description of how to perform the measurements of all disturbances affecting the voltage, hence the PQ, for different classes of devices. Finally, the IEC 61869 series dedicated to ITs contains, in the IEC 61869-6 [54] for the low-power ones, the ratio error and phase displacement limits for all harmonic’s orders. It should be emphasized that such limits can be applied when the ITs are subjected to a single frequency signal and not to a signal consisting of a fundamental component plus several harmonics. However, for the next section, such limits will be used in absence of any other information on the accuracy associated to the harmonic measurements. To this purpose, the limits for ratio error for several accuracy classes are listed in Table 2 for a wide range of frequencies.

Summarizing, the PQ is a quite general terms that include several aspects: (i) limits of the PQ indexes for DSOs, (ii) measuring the electrical quantities when affected by PQ issues, (iii) building measurement devices capable of working under and detect the various meanings of PQ.

However, it is difficult to find some information in the standards regarding the uncertainty related to the PQ measurements, like for the THD, which is the main topic of this work.

## 3. The Closed-Form Expression

### 3.1. Mathematical Development

This section describes how the closed-form expression of the THD uncertainty has been obtained starting from the THD definition. The required input is the accuracy class of the LPIT used to measure the voltage or the current, then the output of the obtained expressions are the mean value and the variance of the THD. For the sake of simplicity, in what follows, the mathematical expressions are given for the voltage case (but they can be replicated also for the current).

Let’s start from the THD definition [51]:

(1) $$THD=\sqrt{\sum_{i=2}^{N}{\left(\frac{V_{i}}{V_{1}}\right)}^{2}},\;$$

where $V_{1}$ is the rms value of the first frequency component of the signal (e.g., 50, 60 Hz), $V_{i}$ is the rms value of the harmonic component of order i, and N is the maximum harmonic order contained in the signal or considered.

The main idea is to rewrite and develop Equation (1) considering the ratio errors affecting the rms values. These are indicated as $\varepsilon_{1}$ and $\varepsilon_{i}$ for $V_{1}$ and $V_{i}$, respectively. Hence:

(2) $$THD=\sqrt{\sum_{i=2}^{N}{\left(\frac{V_{i}\left(1+\varepsilon_{i}\right)}{V_{1}\left(1+\varepsilon_{1}\right)}\right)}^{2}}.$$

The errors $\varepsilon_{1}$ and $\varepsilon_{i}$ assume a value among those listed in Table 2. In particular, $\varepsilon_{1}$ may be one value among those in the second column (dedicated to the 50 Hz component), while $\varepsilon_{i}$ may assume all possible values in the remaining columns, depending on the harmonic order considered (hence, on the frequency).

From the expression of THD in Equation (2), the aim is to exploit the probability distribution of each single element of Equation (2) to obtain the final distribution of THD, of which the mean value $\mu_{THD}$ and the variance $\sigma_{THD}^{2}$ are the desired outcomes. This can be done if, as suggested by the GUM [45], $\varepsilon_{1}$ and $\varepsilon_{i}$ are considered as random variables (r.v.), which varies within the limits listed in Table 2.

The first term that can be analyzed from (2) is:

(3) $$A=V_{i}\left(1+\varepsilon_{i}\right).$$

According to the GUM [45], $\varepsilon_{i}$ can be assumed distributed as a uniform r.v. in the interval $\pm \varepsilon_{i}$. Therefore A is a r.v. uniformly distributed with mean value $\mu_{A}$ and variance $\sigma_{A}^{2}$:

(4) $$\mu_{A}=V_{i},\;$$

(5) $$\sigma_{A}^{2}=V_{i}^{2}\sigma_{\varepsilon h}^{2}.\;$$

In Equation (5), $\sigma_{\varepsilon h}^{2}$ is the variance associated to $\varepsilon_{i}$, hence to the harmonic components (a subscript h has been added for the sake of clarity). The relation between $\sigma_{\varepsilon h}^{2}$ and $\varepsilon_{i}$ is, according to the variance of a uniform distribution:

(6) $$\sigma_{\varepsilon h}^{2}=\frac{{\left(2\varepsilon_{i}\right)}^{2}}{12}.\;$$

Afterwards, in Equation (2) the term A defined in Equation (3) is squared:

(7) $$B={\left[V_{i}\left(1+\varepsilon_{i}\right)\right]}^{2}.\;$$

**Figure 1:**
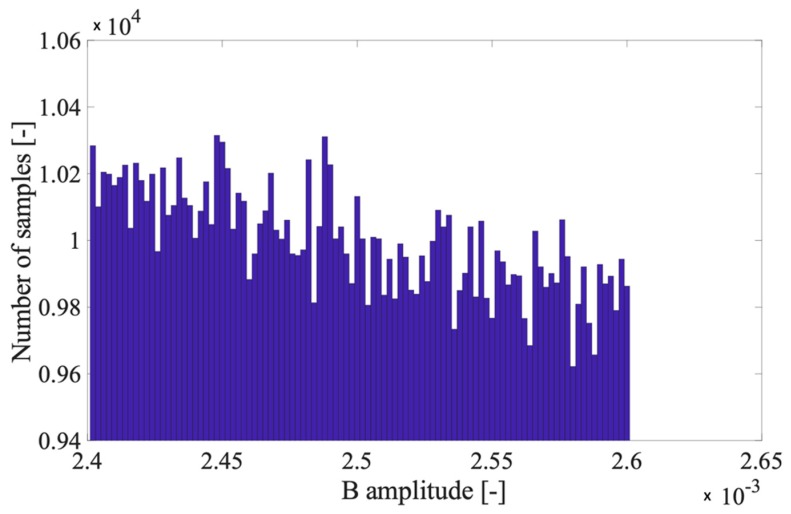
Distribution of B with 1 million trials.

B follows a distribution that is in between a uniform and the inverse of a square root. In particular the probability density function (pdf) of B, $f_{B\left(x\right)}$, is:

(8) $$f_{B\left(x\right)}=\frac{1}{2\varepsilon_{i}\sqrt{x}}\;,\;$$

where x is a generic variable. In Figure 1, the distribution of B is presented for generic values of $V_{i}$ and a million trials.

It is worth clarifying that, in Figure 1, the y axis has been zoomed in on to highlight that B distribution is not fully uniform. In fact, without the zoom, the distribution may appear as uniform. Therefore, from (8), by applying their definition, it is straightforward to obtain the mean value and variance of B:

(9) $$\mu_{B}=V_{i}^{2}+V_{i}\sigma_{\varepsilon h}^{2},\;$$

(10) $$\sigma_{B}^{2}=4V_{i}^{4}\sigma_{\varepsilon h}^{2}+\frac{4}{5}V_{i}^{4}\sigma_{\varepsilon h}^{4}.\;$$

In light of Equations (3)–(10), it is possible to obtain the mean value and variance of the denominator of Equation (2) ($B_{1}={\left[V_{1}\left(1+\varepsilon_{1}\right)\right]}^{2}$) as:

(11) $$\mu_{B1}=V_{1}^{2}+V_{1}\sigma_{\varepsilon }^{2},\;$$

(12) $$\sigma_{B1}^{2}=4V_{1}^{4}\sigma_{\varepsilon }^{2}+\frac{4}{5}V_{1}^{4}\sigma_{\varepsilon }^{4},\;$$

where $\sigma_{\varepsilon }^{2}$ is the variance associated to $\varepsilon_{1}$ that can be easily found applying Equation (6) to $\varepsilon_{1}$.

Turning to the sum of the harmonic components in Equation (2), it is worth noticing that the term $B_{1}$ is independent of it. Therefore, the new term to study can be defined as:

(13) $$C=\sum_{i=2}^{N}{\left[V_{i}\left(1+\varepsilon_{i}\right)\right]}^{2}.\;$$

Such term can be approached using the central limit theorem CLT [55] which guarantees that the mean value and variance of C are the sum of $\mu_{B}$ and $\sigma_{B}^{2}$ for each harmonic order. This results in:

(14) $$\mu_{C}=\sum_{i=2}^{N}\left(V_{i}^{2}+V_{i}\sigma_{\varepsilon h}^{2}\right),\;$$

(15) $$\sigma_{C}^{2}=\sum_{i=2}^{N}\left(4V_{i}^{4}\sigma_{\varepsilon h}^{2}+\frac{4}{5}V_{i}^{4}\sigma_{\varepsilon h}^{4}\right).\;$$

The second to last step before obtaining the mean value and variance of THD is the quotient between C and $B_{1}$:

(16) $$D=\frac{C}{B_{1}}=\frac{\sum_{i=2}^{N}{\left[V_{i}\left(1+\varepsilon_{i}\right)\right]}^{2}}{{\left[V_{1}\left(1+\varepsilon_{1}\right)\right]}^{2}}.\;$$

**Figure 2:**
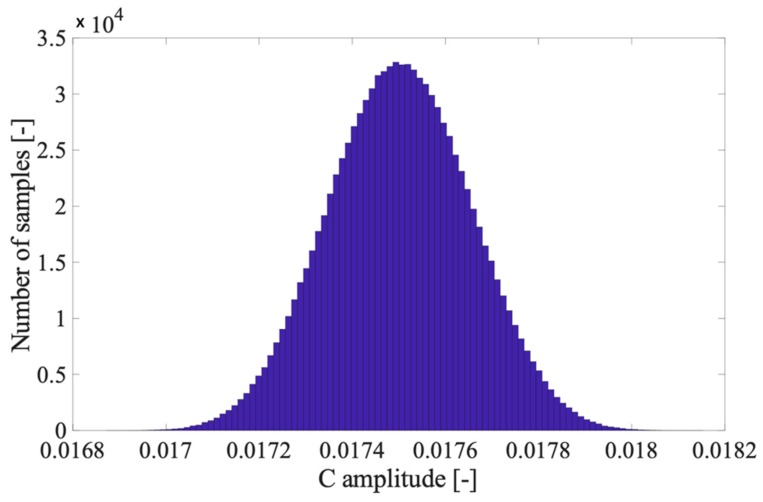
Distribution of C when 5 harmonics with consistent amplitudes are considered.

Its distribution can be obtained considering that:

- C is the sum of several r.v (up to 40 or 50) that can be already assumed normal distributed when five elements are considered. This is confirmed by Figure 2, in which 1 million trials have been run to obtain C using 5 r.v. that satisfy the requirements of the CLT.
- $B_{1}$ is distributed as in Figure 1.

Therefore, the distribution of D results into a quasi-normal distribution with parameters:

(17) $$\mu_{D}=\frac{\mu_{C}}{\mu_{B1}}=\frac{\sum_{i=2}^{N}\left(V_{i}^{2}+V_{i}\sigma_{\varepsilon h}^{2}\right)}{V_{1}^{2}+V_{1}\sigma_{\varepsilon }^{2}},\;$$

(18) $$\sigma_{D}^{2}={\mu_{D}}^{2}\left[{\left(\frac{\sigma_{C}}{\mu_{C}}\right)}^{2}+{\left(\frac{\sigma_{B1}}{\mu_{B1}}\right)}^{2}\right]={\left(\frac{\sum_{i=2}^{N}\left(V_{i}^{2}+V_{i}\sigma_{\varepsilon h}^{2}\right)}{V_{1}^{2}+V_{1}\sigma_{\varepsilon }^{2}}\right)}^{2}\left[\frac{\sum_{i=2}^{N}\left(4V_{i}^{4}\sigma_{\varepsilon h}^{2}+\frac{4}{5}V_{i}^{4}\sigma_{\varepsilon h}^{4}\right)}{{\left(\sum_{i=2}^{N}\left(V_{i}^{2}+V_{i}\sigma_{\varepsilon h}^{2}\right)\right)}^{2}}+\frac{4V_{1}^{4}\sigma_{\varepsilon }^{2}+\frac{4}{5}V_{1}^{4}\sigma_{\varepsilon }^{4}}{{\left(V_{1}^{2}+V_{1}\sigma_{\varepsilon }^{2}\right)}^{2}}\right],$$

where the involved terms are all known from the previous steps.

The definitions of $\mu_{D}$ and $\sigma_{D}^{2}$ have been described in [56] for the ratio of two quasi-normal distributions, with positive and non-zero mean and variance different from one. The assumption that is made in this work is that, considering that C is normally distributed, the weight of $B_{1}$ that is not quasi-normal, in terms of distribution, is not significant compared to C.

**Figure 3:**
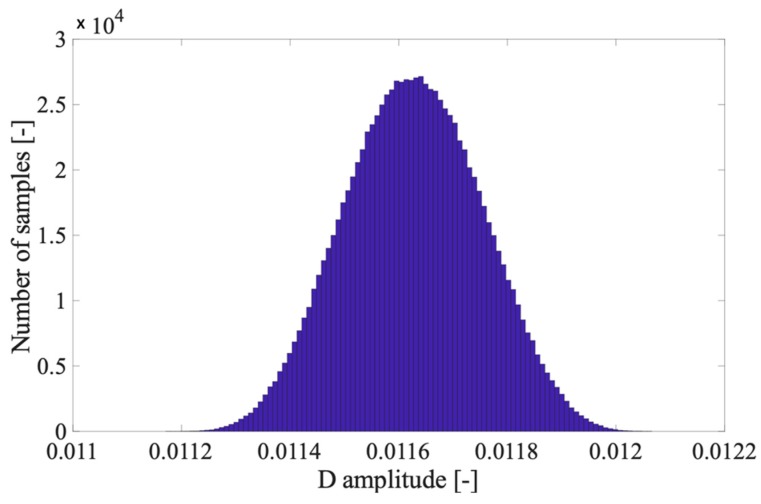
Distribution of D when 5 harmonics with consistent amplitudes are considered.

This aspect is confirmed by Figure 3 where the distribution of D is shown starting from the values used in Figure 2 and running 1 million trials. The relation between $V_{1}$ and all $V_{i}$ has been kept as in a real THD computation: a unity value for $V_{1}$ and the five $V_{i}$ have been randomly chosen from Table 1.

Once D is completely known, it is possible to obtain the desired parameters from (2). In fact, the last step consists of applying the square root to the r.v. D, obtaining the THD.

The r.v. THD follows a Nakagami distribution [57] of parameters m and Ω:

(19) $$m=\frac{\mu_{D}}{\sigma_{D}^{2}},\;$$

(20) $$\Omega =\mu_{D}.\;$$

With the two parameters it is straightforward to compute the mean value and the variance of THD as:

(21) $$\mu_{THD}=P\left(m,\;\frac{1}{2}\right)*\sqrt{\frac{\Omega }{m}},$$

(22) $$\sigma_{THD}^{2}=\Omega \left\{1-\frac{1}{m}{\left[P\left(m,\frac{1}{2}\;\right)\right]}^{2}\right\}.\;$$

In both expressions, P is the well-known Pochhammer function [58] implemented with the parameters m and ½. The latter parameter value is due to presence of only one element under the square root expression of THD, hence to the degrees of freedom.

With Equations (22) and (23), the desired outcome has been achieved: to find closed-form expression for the mean value but most importantly for the variance associated to the THD. Both expressions have as inputs the rms values of the voltage harmonics, the accuracy class of the adopted LPIT and the limits on the harmonics’ uncertainty defined in [54].

The reader must be aware that Equations (21) and (22) are estimates of the THD distribution parameters that are valid under the assumption made in this section. In what follows, the validity of such expressions is numerically proven using several case studies.

### 3.2. Practical Considerations

In light of the overall procedure, it is interesting to link it to some particular aspects of the Standards. Let’s consider the expression of THD in which are involved $\varepsilon_{1}$ and $\varepsilon_{i}$. According to [50], the limits for the harmonics are fixed up to the 25^th^ (1250 Hz). Hence, only the first three columns of ratio errors in Table 2 have to be considered. Note also that there is a ratio 10 between ε for the range 0.1–1 kHz and ε at 50 Hz, while such a ratio increases at 20 for the range 1–1.5 kHz. Furthermore, these ratios are valid for all accuracy classes in Table 2.

Consequentially, two new expressions for the harmonic ratio errors in Equation (2) can be formulated as:

(23) $$\varepsilon_{i}=10\varepsilon_{1},\;\mathrm{for}\;i=2\;\ldots \;20,$$

(24) $$\varepsilon_{i}=20\varepsilon_{1},\;\mathrm{for}\;i=21\;\ldots \;25.$$

Finally, Equation (19) can be rewritten with only one variable, $\varepsilon_{1}$:

(25) $$THD=\sqrt{D}=\sqrt{\frac{\sum_{i=2}^{20}{\left[V_{i}\left(1+10\varepsilon_{1}\right)\right]}^{2}+\sum_{i=21}^{25}{\left[V_{i}\left(1+20\varepsilon_{1}\right)\right]}^{2}}{V_{1}\left(1+\varepsilon_{1}\right)}},$$

which shows that the knowledge of the ratio error of the considered LPIT is sufficient to obtain, by implementing Equation (22), the uncertainty associated to THD.

## 4. Tests & Results

In this section, several tests are presented to assess, first of all, the validity of the presented closed form expression, and second, to understand its peculiarities, limits, and range of applicability.

### 4.1. Validation of the Closed-Form Expression

To validate the closed-form expression, described in Section 3, a case study has to be considered. It involves:

1. Three accuracy classes of LPITs, 0.1, 0.2, and 0.5. This choice has been taken to include a huge variety of devices in the test.
2. Three distorted signals. In the remainder of the work referred to as F, G, and H. The three signals consist of a 50 Hz component plus different harmonic contents. In particular, F contains 4 harmonics, the 2^nd^, 4^th^, 6^th^, and 8^th^; signal G contains 7 harmonics from the 2^nd^ to the 8^th^; finally, signal H contains 15 harmonics from 2^nd^ to the 16^th^. The three signals have been designed to represent various signals with few or several harmonics, even or odd.

The combination of the above conditions leads to 9 test scenarios that have been run by using the Monte Carlo (MC) method in the MatLab environment as a reference for the uncertainty evaluation of THD, as suggested by Supplement 1 of the GUM [59]. In particular, 1 million trials have been run to obtain the reference mean value and variance $\mu_{THD\_c}$ and $\sigma_{THD\_c}^{2}$ of THD.

The input required for this test (and for the following) are (i) the harmonic amplitudes, (ii) the limits for the harmonics’ uncertainty $\varepsilon_{i}$, and (iii) the ratio error $\varepsilon_{1}$. In detail, the harmonic amplitude for signals F, G, and H have been fixed in accordance with Table 1, taking the maximum value for each harmonic and a unity value for the 50 Hz component. As for the accuracy limits, in Table 2 the second and third columns are considered for $\varepsilon_{1}$ and $\varepsilon_{i}$, respectively.

Results of the tests are listed in Table 3. It contains, for the three signals and for each accuracy class (AC), the mean value, variance, and standard deviation of THD, estimated by the closed-form expression ($\mu_{THD\_e}$, $\sigma_{THD\_e}^{2}$, and $\sigma_{THD\_e}$) and the reference provided by the MC simulations ($\mu_{THD\_c}$, $\sigma_{THD\_c}^{2}$, and $\sigma_{THD\_c}$).

Before assessing the results, in Table 3 and in the following the standard deviations $\sigma_{THD\_c}$ and $\sigma_{THD\_e}$ are written with two significant digits to highlight the really small difference (if any) between the references and the results of Equations (21) and (22). Of course, the proper way of representing a standard deviation would have been with one significant digit.

The first general comment from the results is that the presented closed-form expression works. In detail, the difference between $\mu_{THD\_c}$ and $\mu_{THD\_e}$, for all tested cases, is always lower than $10^{-3}$, while the difference between $\sigma_{THD\_c}$ and $\sigma_{THD\_e}$ is always lower than $10^{-5}$. A further comment is possible looking at the relative standard deviation $\varrho_{THD\_e}$ of the estimated values in Table 4, computed as:

(26) $$\varrho_{THD\_e}=\frac{\sigma_{THD\_e}}{\mu_{THD\_e}}.\;$$

In fact, as expected, $\varrho_{THD\_e}$ increases with the accuracy class because, for the lower accurate classes, the limits of accuracy fixed for the harmonics are less stringent (see third column of Table 2).

From Table 3, note that the accuracy class does not influence the correctness of the closed-form expression. A reason for that may be attributed to the weight of the denominator of (25), which include the term related to the AC. In fact, the ratio error of the 50 Hz component is at least one order of magnitude lower compared to the ratio error of each single harmonic component (see Table 2).

A final comment on Table 3 is that the effectiveness of Equations (21) and (22) is not affected by the harmonic content of the signal.

### 4.2. Tests vs. Different Accuracy Limits for Harmonics

This subsection discusses whether the closed-form expressions are affected by the change in the fixed accuracy for the harmonics of the signal. To this purpose, the case study introduced in Section 4.1 has been used to perform two additional tests: (i) one fixing the accuracy limit for all harmonics and all AC at 5%; (ii) another fixing that limit to 0.5%, hence 10 times lower compared to the previous. The results for the tests at 5% and 0.5% are shown in Table 5 and Table 6, respectively, adopting the same notation used for Table 3.

From the two tables, it is clear how the proposed approach is not affected by any value of uncertainty associated to the measurement of the harmonic components, neither the stringent 0.5% nor the quite wide 5%. In addition, the comparison of the two tables provides that, for the same value of THD, the 0.5% limits provide a standard deviation one order of magnitude lower than the one at 5%, showing a linear behavior.

### 4.3. Tests vs. Different Harmonics’ Amplitude

A third interesting test to perform is the verification of whether or not the amplitude of the harmonics influences the closed-form expression proposed. To this purpose, the case study introduced in Section 3 has been applied with some modifications: (i) the uncertainty associated to the harmonic has been kept as defined in Table 2 for each accuracy class; (ii) the amplitude of each single harmonic has been fixed to 5%, 2%, and 0.5% of the 50 Hz component, resulting in three different group of tests. Then, 1 million trials have been run to compare the reference values with those obtained applying (21) and (22). Results are collected in Table 7, Table 8 and Table 9, for the cases at 5%, 2%, and 0.5%, respectively.

From the three tables it can be concluded that also the harmonics’ amplitude does not infer on the applicability of Equations (21) and (22). Furthermore, it is confirmed for all accuracy classes and for different combinations of harmonics (4, 7, and 15 harmonics plus the 50 Hz component). To clarify this conclusion, the relative standard deviation $\varrho_{THD\_e}$ has been calculated—in analogy to Table 4—for the estimated values, in all cases of Table 7, Table 8 and Table 9. Results are listed in Table 10.

From the table it can be highlighted that, for all classes and percentage amplitude of the harmonics, the relative uncertainty is consistent among the considered cases.

### 4.4. Normalized Standard Uncertainty Spread.

This subsection is dedicated to the analysis of the THD uncertainty behavior. In fact, considering that described in the previous subsections, only a few harmonic combinations have been tested so far. To this purpose, the closed-form expression has been applied to the following case study: (i) the three AC used above, and signals F, G, and H, (ii) harmonic components amplitude that can vary between zero and the maximum values listed in Table 1; (iii) accuracy of the harmonic component amplitudes fixed at those listed in Table 2.

Hence, 10,000 trials, in which only the harmonics’ amplitude varied as in (ii), have been run and the maximum and minimum values of $\varrho_{THD\_e}$ have been extracted and collected, as shown in Table 11.

**Figure 4:**
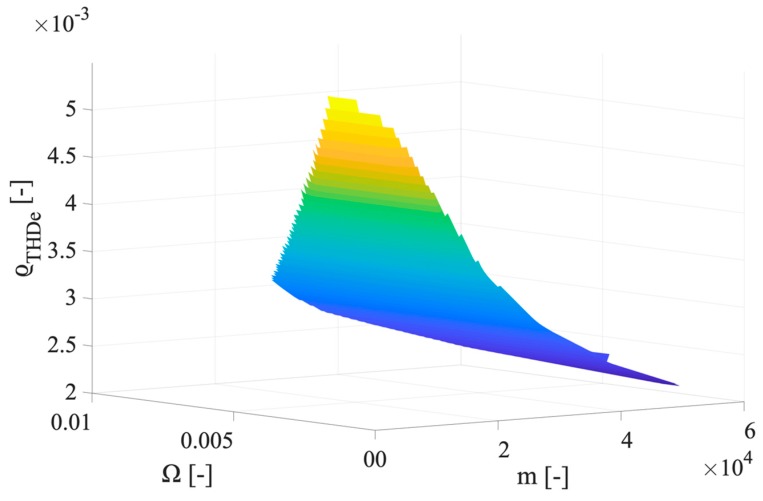
Graph of $\varrho_{THD\_e}$ values vs. m and Ω, for the accuracy class 0.1.

**Figure 5:**
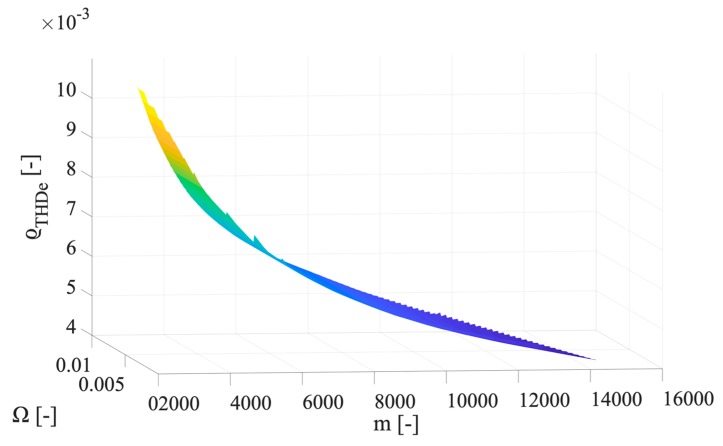
Graph of $\varrho_{THD\_e}$ values vs. m and Ω, for the accuracy class 0.2.

**Figure 6:**
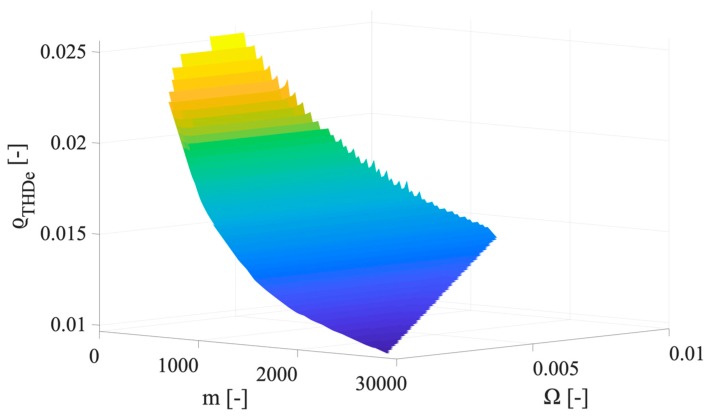
Graph of $\varrho_{THD\_e}$ values vs. m and Ω, for the accuracy class 0.5.

As it can be seen from the table, the max $\varrho_{THD\_e}$ of the classes 0.1, 0.2, and 0.5, never exceeds in percentage 0.58%, 1.2%, and 2.8%, respectively. Furthermore, the range of $\varrho_{THD\_e}$ variation is quite wide; hence, it is strictly dependent on the parameters adopted for its computations: m and Ω. To this purpose, in Figure 4, Figure 5 and Figure 6 the $\varrho_{THD\_e}$ values are plotted vs. m and Ω, for the accuracy class 0.1, 0.2, and 0.5. For the sake of brevity, the three graphs represent the case with 15 harmonics components, hence signal H.

For the sake of readability, the three above graphs have been appositely presented with different inclinations of the surface. This way, it is possible to better appreciate how $\varrho_{THD\_e}$ is affected by m and Ω. The main conclusion by inspecting the surfaces is that high values of m are associated to low values of $\varrho_{THD\_e}$, which is reasonable in light of Equation (20). In fact, low values of m can be obtained when the variance used for its computation is low.

### 4.5. Tests vs. High Number of Harmonic Components

This subsection ends the test section with a focus on the practical consideration introduced in Section 3.2. In particular, the first part is dedicated to assess the validity of the closed-form expression in the worst cases of 20 and 25 harmonic components; while the second part tackles the possibility of simplify what presented in Section 3.2.

#### 4.5.1. Tests with 20 and 25 Harmonic Components

Two new signals have been designed to test in even worse cases the proposed approach. Signal I consists of a fundamental component plus all harmonics from the 2^nd^ to the 20^th^, while signal L is equal to I but with harmonic up to the 25^th^. Again, as in Section 4.1, the accuracy associated to the harmonics are those in Table 2, while their amplitudes are the maximum value listed in Table 1. It is worth to highlight that, for the case of signal L the accuracy limit of the harmonic component from the 21^st^ to the 25^th^ is different from the one of the first 20 components.

With the same notation as Table 3, Table 12 contains the mean value, variance, and standard deviation of THD, estimated by the closed-form expression ($\mu_{THD\_e}$, $\sigma_{THD\_e}^{2}$, and $\sigma_{THD\_e}$) and obtained by the MC trials ($\mu_{THD\_c}$, $\sigma_{THD\_c}^{2}$, and $\sigma_{THD\_c}$).

From the figures in the table, the effectiveness of the closed-form expression is confirmed. Again, the uncertainty associated to THD decreases with the less stringent AC in both cases of signal I and L. Furthermore, it is possible to conclude that the proposed approach covers all harmonic components tackled in [50], hence it is suitable for DSO’s applications.

#### 4.5.2. A Further Simplification of the Expression

In light of Section 4.5.1, the closed-form expression is effective in all the possible operating conditions that may rise from [50]. Equation (25) showed that, by considering the limits defined by the standard for the different ACs, the expression of THD as function of ratio errors only depends on $\varepsilon_{1}$. However, for each range of frequency, a different coefficient (10 or 20) has to be applied to $\varepsilon_{1}$. Therefore, this subsection aims at proving that Equation (25), written by using only the coefficient 10 for all harmonics from the 2^nd^ to the 25^th^ (with amplitudes in accordance with EN 50160 [50]), leads to values of $\mu_{THD\_e}$ and $\sigma_{THD\_e}$ consistent with those obtained starting from Equation (25). To prove that, what is tested in Section 4.5.1 is replicated adopting the limits given in column 3 of Table 2 for all harmonic components up to the 25^th^ (1250 Hz).

Therefore, 1 million trials have been performed with signal L and all classes. The results are presented in Table 13.

Values in Table 13 can be directly compared with those in Table 12 for signal L. The comparison confirms that it is possible to assume the same uncertainty limit for the harmonics, without distinction, from the 2^nd^ to the 25^th^. In fact, considering that two significant digits are presented only for the sake of comparison, the standard deviations in Table 12 and Table 13 are identical. Hence, there is no missing information from:

(27) $$THD=\sqrt{\frac{\sum_{i=2}^{25}{\left[V_{i}\left(1+10\varepsilon_{1}\right)\right]}^{2}}{V_{1}\left(1+\varepsilon_{1}\right)}},$$

for the computation of $\mu_{THD\_e}$ and $\sigma_{THD\_e}$, with respect to those obtained starting from (25). In other words, this leads to conclude that, the contribution to the THD uncertainty given by the harmonic components from the 21^st^ to the 25^th^ is negligible if the limits of [50] are met.

## 5. Conclusions

In this work is presented a closed-form expression to estimate the mean value and variance of the well-known power quality parameter measured by means of an LPIT, the THD. After describing the mathematical steps required to obtain the expressions, several tests have been run to (i) verify their correctness and applicability, (ii) to stress them to understand their limits and peculiarities. The test results confirm the effectiveness and accuracy of the closed-form expression to evaluate the mean value and variance of THD. Such values can be obtained for signals containing all harmonic components considered by the standards and in all range of amplitudes. The proposed approach has also been stressed to obtain a simplified expression to calculate the uncertainty of THD which only requires the knowledge of the ratio error of the instrument transformer involved.

Overall, the results of this study may be of great help in particular for DSOs and utilities operators to estimate, at a glance, the uncertainty related to their THD measurements.

## Figures and Tables

**Table 1:** Values of the harmonic limits at the supply terminals for orders up to 25^th.^

Odd Harmonics				Even Harmonics	
Not Multiples of 3		Multiples of 3			
Order h	Relative Amplitude $u_{h}$	Order h	Relative Amplitude $u_{h}$	Order h	Relative Amplitude $u_{h}$
3	6.0%	3	5.0%	2	2.0%
7	5.0%	9	1.5%	4	1.0%
11	3.5%	15	0.5%	6 to 24	0.5%
13	3.0%	21	0.5%		
17	2.0%				
19	1.5%				
23	1.5%				
25	1.5%				

**Table 2:** Ratio error for each accuracy class and for different range of frequencies.

Accuracy Class	Ratio Error ε [%]			
	50 Hz	0.1 to 1 kHz	1 to 1.5 kHz	1.5 to 3 kHz
**0.1**	±0.1	±1	±2	±5
**0.2**	±0.2	±2	±4	±5
**0.5**	±0.5	±5	±10	±10
**1**	±1	±10	±20	±20

**Table 3:** Results of the validation test, which involves three signals and 3 accuracy classes.

Signal	AC	$\mu_{THD\_c}$ (-)	$\mu_{THD\_e}$ (-)	$\sigma_{THD\_c}^{2}$ (-)	$\sigma_{THD\_e}^{2}$ (-)	$\sigma_{THD\_c}$ (-)	$\sigma_{THD\_e}$ (-)
**F**	0.1	0.0235	0.0235	1.06 $\times \;10^{-8}$	1.05 $\times \;10^{-8}$	1.0 $\times \;10^{-4}$	1.0 $\times \;10^{-4}$
	0.2	0.0235	0.0236	4.23 $\times \;10^{-8}$	4.19 $\times \;10^{-8}$	2.1 $\times \;10^{-4}$	2.0 $\times \;10^{-4}$
	0.5	0.0235	0.0241	2.64 $\times \;10^{-8}$	2.49 $\times \;10^{-8}$	5.1 $\times \;10^{-4}$	5.0 $\times \;10^{-4}$
**G**	0.1	0.096	0.096	9.66 $\times \;10^{-8}$	9.64 $\times \;10^{-8}$	3.1 $\times \;10^{-4}$	3.1 $\times \;10^{-4}$
	0.2	0.0957	0.0958	3.86 $\times \;10^{-8}$	3.85 $\times \;10^{-7}$	6.2 $\times \;10^{-4}$	6.2 $\times \;10^{-4}$
	0.5	0.096	0.097	2.41 $\times \;10^{-6}$	2.37 $\times \;10^{-6}$	1.6 $\times \;10^{-3}$	1.5 $\times \;10^{-3}$
**H**	0.1	0.108	0.108	8.43 $\times \;10^{-8}$	8.41 $\times \;10^{-8}$	2.9 $\times \;10^{-4}$	2.9 $\times \;10^{-4}$
	0.2	0.1078	0.1080	3.37 $\times \;10^{-7}$	3.36 $\times \;10^{-7}$	5.8 $\times \;10^{-4}$	5.8 $\times \;10^{-4}$
	0.5	0.108	0.109	2.10 $\times \;10^{-6}$	2.06 $\times \;10^{-6}$	1.4 $\times \;10^{-3}$	1.4 $\times \;10^{-3}$

**Table 4:** Relative standard deviation of the estimated values.

Signal	AC	$\varrho_{THD\_e}$ (-)
**F**	0.1	4.4 $\times \;10^{-3}$
	0.2	8.7 $\times \;10^{-3}$
	0.5	2.1 $\times \;10^{-2}$
**G**	0.1	3.2 $\times \;10^{-3}$
	0.2	$6.5\times \;10^{-3}$
	0.5	1.6 $\times \;10^{-2}$
**H**	0.1	2.7 $\times \;10^{-3}$
	0.2	5.4 $\times \;10^{-3}$
	0.5	1.3 $\times \;10^{-2}$

**Table 5:** Results of the test vs. harmonic accuracy class limit at 5%.

Signal	AC	$\mu_{THD\_c}$ (-)	$\mu_{THD\_e}$ (-)	$\sigma_{THD\_c}^{2}$ (-)	$\sigma_{THD\_e}^{2}$ (-)	$\sigma_{THD\_c}$ (-)	$\sigma_{THD\_e}$ (-)
**F**	0.1	0.0235	0.0241	2.60 $\times \;10^{-7}$	2.45 $\times \;10^{-7}$	5.1 $\times \;10^{-4}$	4.9 $\times \;10^{-4}$
	0.2	0.0235	0.0241	2.60 $\times \;10^{-7}$	2.45 $\times \;10^{-7}$	5.1 $\times \;10^{-4}$	5.0 $\times \;10^{-4}$
	0.5	0.0235	0.0241	2.64 $\times \;10^{-7}$	2.49 $\times \;10^{-7}$	5.1 $\times \;10^{-4}$	5.0 $\times \;10^{-4}$
**G**	0.1	0.096	0.097	2.34 $\times \;10^{-6}$	2.30 $\times \;10^{-6}$	1.5 $\times \;10^{-3}$	1.5 $\times \;10^{-3}$
	0.2	0.096	0.097	2.34 $\times \;10^{-6}$	2.31 $\times \;10^{-6}$	1.5 $\times \;10^{-3}$	1.5 $\times \;10^{-3}$
	0.5	0.096	0.097	2.41 $\times \;10^{-6}$	2.37 $\times \;10^{-6}$	1.6 $\times \;10^{-3}$	1.5 $\times \;10^{-3}$
**H**	0.1	0.108	0.109	2.01 $\times \;10^{-6}$	1.97 $\times \;10^{-6}$	1.4 $\times \;10^{-3}$	1.4 $\times \;10^{-3}$
	0.2	0.108	0.109	2.02 $\times \;10^{-6}$	1.98 $\times \;10^{-6}$	1.4 $\times \;10^{-3}$	1.4 $\times \;10^{-3}$
	0.5	0.108	0.109	2.10 $\times \;10^{-6}$	2.06 $\times \;10^{-6}$	1.4 $\times \;10^{-3}$	1.4 $\times \;10^{-3}$

**Table 6:** Results of the test vs. harmonic accuracy class limit at 0.5%.

Signal	AC	$\mu_{THD\_c}$ (-)	$\mu_{THD\_e}$ (-)	$\sigma_{THD\_c}^{2}$ (-)	$\sigma_{THD\_e}^{2}$ (-)	$\sigma_{THD\_c}$ (-)	$\sigma_{THD\_e}$ (-)
**F**	0.1	0.02345	0.02346	2.78 $\times \;10^{-9}$	2.78 $\times \;10^{-9}$	5.3 $\times \;10^{-5}$	5.3 $\times \;10^{-5}$
	0.2	0.02345	0.02346	3.33 $\times \;10^{-9}$	3.33 $\times \;10^{-9}$	5.8 $\times \;10^{-5}$	5.8 $\times \;10^{-5}$
	0.5	0.02345	0.02346	7.16 $\times \;10^{-9}$	7.18 $\times \;10^{-9}$	8.5 $\times \;10^{-5}$	8.5 $\times \;10^{-5}$
**G**	0.1	0.0957	0.0957	2.64 $\times \;10^{-8}$	2.64 $\times \;10^{-8}$	1.6 $\times \;10^{-4}$	1.6 $\times \;10^{-4}$
	0.2	0.0957	0.0957	3.55 $\times \;10^{-8}$	3.55 $\times \;10^{-8}$	1.9 $\times \;10^{-4}$	1.9 $\times \;10^{-4}$
	0.5	0.0957	0.0957	9.95 $\times \;10^{-8}$	9.96 $\times \;10^{-8}$	3.2 $\times \;10^{-4}$	3.2 $\times \;10^{-4}$
**H**	0.1	0.1078	0.1078	2.39 $\times \;10^{-8}$	2.39 $\times \;10^{-8}$	1 $\times \;10^{-4}$.	1.5 $\times \;10^{-4}$
	0.2	0.1078	0.1078	3.55 $\times \;10^{-8}$	3.56 $\times \;10^{-8}$	1.9 $\times \;10^{-4}$	1.9 $\times \;10^{-4}$
	0.5	0.1078	0.1078	1.17 $\times \;10^{-7}$	1.17 $\times \;10^{-7}$	3.4 $\times \;10^{-4}$	3.4 $\times \;10^{-4}$

**Table 7:** Results of the test vs. harmonic amplitude, all at 5% of the 50 Hz component.

Signal	AC	$\mu_{THD\_c}$ (-)	$\mu_{THD\_e}$ (-)	$\sigma_{THD\_c}^{2}$ (-)	$\sigma_{THD\_e}^{2}$ (-)	$\sigma_{THD\_c}$ (-)	$\sigma_{THD\_e}$ (-)
**F**	0.1	0.100	0.100	8.65 $\times \;10^{-8}$	8.66 $\times \;10^{-8}$	2.9 $\times \;10^{-4}$	2.9 $\times \;10^{-4}$
	0.2	0.100	0.100	3.47 $\times \;10^{-7}$	3.46 $\times \;10^{-7}$	5.9 $\times \;10^{-4}$	5.9 $\times \;10^{-4}$
	0.5	0.100	0.101	2.17 $\times \;10^{-6}$	2.13 $\times \;10^{-6}$	1.5 $\times \;10^{-3}$	1.5 $\times \;10^{-3}$
**G**	0.1	0.132	0.132	8.90 $\times \;10^{-8}$	8.91 $\times \;10^{-8}$	3.0 $\times \;10^{-4}$	3.0 $\times \;10^{-4}$
	0.2	0.132	0.132	3.57 $\times \;10^{-7}$	3.56 $\times \;10^{-7}$	6.0 $\times \;10^{-4}$	6.0 $\times \;10^{-4}$
	0.5	0.132	0.133	2.23 $\times \;10^{-6}$	2.20 $\times \;10^{-6}$	1.5 $\times \;10^{-3}$	1.5 $\times \;10^{-3}$
**H**	0.1	0.194	0.194	9.59 $\times \;10^{-8}$	9.58 $\times \;10^{-8}$	3.1 $\times \;10^{-4}$	3.1 $\times \;10^{-4}$
	0.2	0.1937	0.1939	3.84 $\times \;10^{-7}$	3.83 $\times \;10^{-7}$	6.2 $\times \;10^{-4}$	6.2 $\times \;10^{-4}$
	0.5	0.194	0.195	2.40 $\times \;10^{-6}$	2.37 $\times \;10^{-6}$	1.5 $\times \;10^{-3}$	1.5 $\times \;10^{-3}$

**Table 8:** Results of the test vs. harmonic amplitude, all at 2% of the 50 Hz component.

Signal	AC	$\mu_{THD\_c}$ (-)	$\mu_{THD\_e}$ (-)	$\sigma_{THD\_c}^{2}$ (-)	$\sigma_{THD\_e}^{2}$ (-)	$\sigma_{THD\_c}$ (-)	$\sigma_{THD\_e}$ (-)
**F**	0.1	0.0400	0.0400	1.38 $\times \;10^{-8}$	1.38 $\times \;10^{-8}$	1.2 $\times \;10^{-4}$	1.2 $\times \;10^{-4}$
	0.2	0.0400	0.0401	5.56 $\times \;10^{-8}$	5.51 $\times \;10^{-8}$	2.4 $\times \;10^{-4}$	2.3 $\times \;10^{-4}$
	0.5	0.0400	0.0408	3.47 $\times \;10^{-7}$	3.34 $\times \;10^{-7}$	5.9 $\times \;10^{-4}$	5.8 $\times \;10^{-4}$
**G**	0.1	0.0529	0.0530	1.42 $\times \;10^{-8}$	1.42 $\times \;10^{-8}$	1.2 $\times \;10^{-4}$	1.2 $\times \;10^{-4}$
	0.2	0.0529	0.0531	5.72 $\times \;10^{-8}$	5.67 $\times \;10^{-8}$	2.4 $\times \;10^{-4}$	2.4 $\times \;10^{-4}$
	0.5	0.0529	0.0540	3.57 $\times \;10^{-7}$	3.44 $\times \;10^{-7}$	6.0 $\times \;10^{-4}$	5.9 $\times \;10^{-4}$
**H**	0.1	0.077	0.078	1.53 $\times \;10^{-8}$	1.53 $\times \;10^{-8}$	1.2 $\times \;10^{-4}$	1.2 $\times \;10^{-4}$
	0.2	0.0775	0.0777	6.13 $\times \;10^{-8}$	6.10 $\times \;10^{-8}$	2.5 $\times \;10^{-4}$	2.5 $\times \;10^{-4}$
	0.5	0.077	0.079	3.84 $\times \;10^{-7}$	3.72 $\times \;10^{-7}$	6.2 $\times \;10^{-4}$	6.1 $\times \;10^{-4}$

**Table 9:** Results of the test vs. harmonic amplitude, all at 0.5% of the 50 Hz component.

Signal	AC	$\mu_{THD\_c}$ (-)	$\mu_{THD\_e}$ (-)	$\sigma_{THD\_c}^{2}$ (-)	$\sigma_{THD\_e}^{2}$ (-)	$\sigma_{THD\_c}$ (-)	$\sigma_{THD\_e}$ (-)
**F**	0.1	0.0100	0.0100	8.68 $\times \;10^{-10}$	8.61 $\times \;10^{-10}$	2.9 $\times \;10^{-5}$	2.9 $\times \;10^{-5}$
	0.2	0.0100	0.0101	3.46 $\times \;10^{-9}$	3.38 $\times \;10^{-9}$	5.9 $\times \;10^{-5}$	5.8 $\times \;10^{-5}$
	0.5	0.0100	0.0108	2.17 $\times \;10^{-8}$	1.88 $\times \;10^{-8}$	1.5 $\times \;10^{-4}$	1.4 $\times \;10^{-4}$
**G**	0.1	0.0132	0.0133	8.91 $\times \;10^{-10}$	8.87 $\times \;10^{-10}$	3.0 $\times \;10^{-5}$	3.0 $\times \;10^{-5}$
	0.2	0.0132	0.0134	3.56 $\times \;10^{-9}$	3.49 $\times \;10^{-9}$	6.0 $\times \;10^{-5}$	5.9 $\times \;10^{-5}$
	0.5	0.0132	0.0143	2.23 $\times \;10^{-8}$	1.96 $\times \;10^{-8}$	1.5 $\times \;10^{-4}$	1.4 $\times \;10^{-4}$
**H**	0.1	0.019	0.019	9.59 $\times \;10^{-10}$	9.54 $\times \;10^{-10}$	3.1 $\times \;10^{-5}$	3.1 $\times \;10^{-5}$
	0.2	0.0194	0.0196	3.83 $\times \;10^{-9}$	3.76 $\times \;10^{-9}$	6.2 $\times \;10^{-5}$	6.1 $\times \;10^{-5}$
	0.5	0.019	0.021	2.39 $\times \;10^{-8}$	2.15 $\times \;10^{-8}$	1.5 $\times \;10^{-4}$	1.5 $\times \;10^{-4}$

**Table 10:** Relative standard deviation of the estimated values.

Signal	AC	5% Amplitude	2% Amplitude	0.5% Amplitude
		$\varrho_{THD\_e}$ (-)	$\varrho_{THD\_e}$ (-)	$\varrho_{THD\_e}$ (-)
**F**	0.1	2.9 $\times \;10^{-3}$	2.9 $\times \;10^{-3}$	2.9 $\times \;10^{-3}$
	0.2	5.9 $\times \;10^{-3}$	5.9 $\times \;10^{-3}$	5.7 $\times \;10^{-3}$
	0.5	1.4 $\times \;10^{-2}$	1.4 $\times \;10^{-2}$	1.3 $\times \;10^{-2}$
**G**	0.1	2.3 $\times \;10^{-3}$	2.3 $\times \;10^{-3}$	2.2 $\times \;10^{-3}$
	0.2	$4.5\times 10^{-3}$	$4.5\times 10^{-3}$	$4.4\times 10^{-3}$
	0.5	1.1 $\times \;10^{-2}$	1.1 $\times \;10^{-2}$	9.8 $\times \;10^{-3}$
**H**	0.1	1.6 $\times \;10^{-3}$	1.6 $\times \;10^{-3}$	1.6 $\times \;10^{-3}$
	0.2	3.2 $\times \;10^{-3}$	3.2 $\times \;10^{-3}$	3.1 $\times \;10^{-3}$
	0.5	7.9 $\times \;10^{-3}$	7.7 $\times \;10^{-3}$	7.0 $\times \;10^{-3}$

**Table 11:** Min and max values of $\varrho_{THD\_e}$ for the three signals and all accuracy classes.

Signal	AC	$\varrho_{THD\_e}$	
		Min (-)	Max (-)
**F**	0.1	2.9 $\times \;10^{-3}$	5.8 $\times \;10^{-3}$
	0.2	5.5 $\times \;10^{-3}$	1.2 $\times \;10^{-2}$
	0.5	9.0 $\times \;10^{-3}$	2.8 $\times \;10^{-2}$
**G**	0.1	2.6 $\times \;10^{-3}$	5.7 $\times \;10^{-3}$
	0.2	5.0 $\times \;10^{-3}$	1.1 $\times \;10^{-2}$
	0.5	1.0 $\times \;10^{-2}$	2.8 $\times \;10^{-2}$
**H**	0.1	2.2 $\times \;10^{-3}$	5.5 $\times \;10^{-3}$
	0.2	4.2 $\times \;10^{-3}$	1.1 $\times \;10^{-2}$
	0.5	9.6 $\times \;10^{-3}$	2.6 $\times \;10^{-2}$

**Table 12:** Results of test, for signals *I* and *L*, for the 3 accuracy classes.

Signal	AC	$\mu_{THD\_c}$ (-)	$\mu_{THD\_e}$ (-)	$\sigma_{THD\_c}^{2}$ (-)	$\sigma_{THD\_e}^{2}$ (-)	$\sigma_{THD\_c}$ (-)	$\sigma_{THD\_e}$ (-)
**I**	0.1	0.1109	0.1110	8.07 $\times \;10^{-8}$	8.05 $\times \;10^{-8}$	2.8 $\times \;10^{-4}$	2.8 $\times \;10^{-4}$
	0.2	0.1109	0.1111	3.22 $\times \;10^{-7}$	3.21 $\times \;10^{-7}$	5.7 $\times \;10^{-4}$	5.7 $\times \;10^{-4}$
	0.5	0.111	0.112	2.01 $\times \;10^{-6}$	1.97 $\times \;10^{-6}$	1.4 $\times \;10^{-3}$	1.4 $\times \;10^{-3}$
**L**	0.1	0.1132	0.1133	7.89 $\times \;10^{-8}$	7.86 $\times \;10^{-8}$	2.8 $\times \;10^{-4}$	2.8 $\times \;10^{-4}$
	0.2	0.1133	0.1136	3.14 $\times \;10^{-7}$	3.13 $\times \;10^{-7}$	5.6 $\times \;10^{-4}$	5.6 $\times \;10^{-4}$
	0.5	0.113	0.115	1.97 $\times \;10^{-6}$	1.91 $\times \;10^{-6}$	1.4 $\times \;10^{-3}$	1.4 $\times \;10^{-3}$

**Table 13:** Results of test, for signal *L* and for the 3 accuracy classes, with the simplified expression.

Signal	AC	$\mu_{THD\_c}$ (-)	$\mu_{THD\_e}$ (-)	$\sigma_{THD\_c}^{2}$ (-)	$\sigma_{THD\_e}^{2}$ (-)	$\sigma_{THD\_c}$ (-)	$\sigma_{THD\_e}$ (-)
**L**	0.1	0.1132	0.1133	7.78 $\times \;10^{-8}$	7.78 $\times \;10^{-8}$	2.8 $\times \;10^{-4}$	2.8 $\times \;10^{-4}$
	0.2	0.1133	0.1135	3.11 $\times \;10^{-7}$	3.10 $\times \;10^{-7}$	5.6 $\times \;10^{-4}$	5.6 $\times \;10^{-4}$
	0.5	0.113	0.115	1.95 $\times \;10^{-6}$	1.90 $\times \;10^{-6}$	1.4 $\times \;10^{-3}$	1.4 $\times \;10^{-3}$
